# The reactive element effect of ceria particle dispersion on alumina growth: A model based on microstructural observations

**DOI:** 10.1038/srep29593

**Published:** 2016-07-13

**Authors:** X. Wang, X. Peng, X. Tan, F. Wang

**Affiliations:** 1 School of Materials Science and Engineering, University of Science and Technology of China, Hefei, 230026, China; 2 Laboratory for Corrosion and Protection, Institute of Metal Research, Chinese Academy of Sciences, Shenyang, 110016, China

## Abstract

The oxidation kinetics of alumina-forming metals can be affected by adding a small amount of a reactive (normally rare earth) element oxide (RE_x_O_y_) and the segregation of the reactive element (RE) ions to the growing alumina grain boundaries (GBs) has been considered as a responsible reason. However, this interpretation remains a controversial issue as to how RE ions are produced by RE_x_O_y_ which is thermodynamically and chemically stable in metals. The question is answered by a model that is based on transmission electron microscopy (TEM) investigation of a CeO_2_-dispersed nickel aluminide oxidized in air at 1100 °C. The CeO_2_ dispersion is incorporated into the alumina scale by the inward growth of inner α-Al_2_O_3_, where it partially dissolves producing tetravalent Ce cations which then transform to trivalent cations by trapping electrons. The trivalent cations segregate to the α-Al_2_O_3_ GBs and diffuse outward along first the GBs and later the twin boundaries (TBs) in the outer γ-Al_2_O_3_ layer, being precipitated as Ce_2_O_3_ particles near surface.

High temperature oxidation, a thermally- and chemically-activated reaction process with an expected increase in severity as the temperature increases, is a key mode of environmental degradation of high temperature structural materials. It normally leads to a loss of their service life. The service life can, however, be highly prolonged if the materials have the ability to develop a scale of thermally grown oxide (TGO) with the merits of compactness, slow growth and thermodynamic stability. The α-Al_2_O_3_ TGO is such a representative oxide, which can offer excellent resistance to oxidation above 1000 °C. Development and study of oxidation-resistant alumina formers at high temperatures has been attracting great interests for decades. Alumina-forming MCrAl system (M = Ni, Fe, Co, or their combinations) and nickel aluminides are the well-known oxidation-resistant alloys and coatings. Many reports show that these alumina formers can be further improved in the high temperature oxidation performance by adding small amounts of reactive elements such as yttrium, hafnium, cerium, zirconium, and lanthanum^1^,^2^,^3^,^4^,^5^,^6^,^7^,^8^,^9^,^10^,^11^,^12^,^13^,^14^,^15^,^16^,^17^,^18^,^19^,^20^,^21^,^22^,^23^,^24^,^25^,^26^,^27^,^28^,^29^,^30^,^31^,^32^,^33^,^34^,^35^,^36^,^37^,^38^,^39^.

The phenomenon that the REs additions improve the oxidation resistance of metals was first found in 1937 by Pfeil^40^ and is popularly referred to as the “reactive element effects (REEs)”. The RE additions into alumina formers are conventionally made by alloying^1^,^4^,^5^,^7^,^8^,^12^,^13^,^15^,^19^,^22^,^23^,^25^,^26^,^27^,^34^,^35^,^39^, ion implantation^5^,^8^,^9^,^11^,^15^,^29^,^31^,^35^ and RE_x_O_y_ dispersions^2^,^3^,^6^,^7^,^10^,^14^,^15^,^17^,^18^,^20^,^24^,^28^,^32^,^33^. The REEs on alumina formers have been summarized and reviewed successively by some authors^41^,^42^,^43^,^44^,^45^,^46^.

One typical REE for alumina formers is that the RE addition tends to decrease their oxidation rates. For example, a decrease in the alumina TGO growth rate has been observed in RE-implanted^8^,^9^,^15^,^29^ and RE-alloyed^8^,^12^,^15^,^38^ β-NiAl. This aspect of REE have been interpreted by two popular models. One is a so-called poisoned interface model (PIM)^47^, which applies well to interpretation of the REE on growth of the cation-diffusion oxide (e.g. chromia^48^) scale. In the PIM, RE atoms are proposed to segregate to the scale/metal interface, pinning the climb of misfit dislocations there required for the scale growth. The other is the grain boundary (GB) segregation model. It indicates that the RE ions which incorporate into the growing alumina normally tend to segregate to the oxide GBs, where they exert the REE through either “site blocking” − blocking the fast diffusion paths for Al^3+^^3^,^6^,^15^, or a “swamping-out” mechanism^49^, in which the isovalent segregants (e.g. Y^3+^) suppress segregation of other divalent and tetravalent cations (e.g., Ca^2+^ and Si^4+^^50^). The aliovalent cation segregation in alumina can enhance the GB diffusivity of aluminum cations (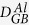) and oxygen anions (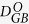) by increasing the number of anion and cation vacancies. More recently, some investigators attributed the REE on the alumina growth to a modification of the electronic structure of alumina with GB donor and acceptor states to the extent that Al ionization at the interface is decreased^30^,^36^.

The RE segregation model has been supported by many experimental observations of RE segregation at the alumina GBs by means of TEM in a combination of X-ray energy-dispersve spectroscopy (EDS)^3^,^4^,^6^,^7^,^10^,^12^,^15^,^17^,^32^. In addition to the GB segregation, RE has been found to occur as RE_x_O_y_ particles on the alumina scale surface^15^,^17^. On its basis, a dynamic segregation theory (DST) has been proposed, in which the RE segregants at the GBs are not static; they can transport outward along the GBs driven by the oxygen potential (i.e., oxygen chemical activity) gradient across a growing alumina scale and their high affinity for oxygen^15^.

Many literatures^15^,^17^,^20^,^24^,^25^,^28^ also reported that addition of the RE_x_O_y_ dispersions in an alloy plays a similar role as RE in decreasing the oxidation rate of alumina-forming metals. This effectiveness REE of the RE_x_O_y_ on the alumina TGO growth is firstly attributed to the dissolution of RE_x_O_y_ which is proposed to occur under the oxygen pressure gradient across the metal-oxide-gas system^15^,^24^, producing RE atoms to segregate to the oxide/metal interface. However, there has been no experimental evidence for such dissolution (or dissociation) and the latter also appears to have no thermodynamic justification, because RE_x_O_y_ (more stable than Al_2_O_3_) have a very high thermal stability. The oxygen pressure in the metals, which decreases from the dissociation pressure of Al_2_O_3_ at the interface to some low values (depending on the oxygen solubility and diffusivity) at some distance from the interface, is normally not low enough to drive the RE_x_O_y_ dispersions to dissociate and release RE atoms which can segregate to the interface. In view of this, the concerned REE exerted by RE_x_O_y_ appears not be explained fully by DST, although it cannot be explained appropriately by PIM. A much more likelihood that the RE_x_O_y_ dispersions exert the REE is associated with the dissolution of RE_x_O_y_ dispersions upon incorporation into the alumina scales, as described simply in^3^,^6^. This raises a question on how the oxide dispersions enter the alumina TGO.

Recently, an interpretation on the effect of the ceria particle dispersion on the growth process of alumina scale on an alumina-forming aluminide was proposed^32^. It highlights two points. First, the incorporation of the ceria particles into the alumina TGO results from inward growth of the inner part of the alumina in α phase. Second, the ceria particles do not exhibit the REE until they have been incorporated into the alumina scale, where they may dissolve to some extent to produce the cerium ions that can segregate to the alumina GBs and thus suppress the outward diffusion of Al cations along the short-circuit paths for the TGO growth. However, this interpretation is still lack of sufficient evidences. We further characterized the microstructure of the alumina TGO on the ceria-dispersed aluminide and traced cerium either in its elementary or oxide form from the metal to the TGO. There are new observations: (i) no dissolution of original CeO_2_ particles in the metal during oxidation, (ii) the identification that the TGO scale is composed of the outward growing γ-Al_2_O_3_ and inward growing α-Al_2_O_3_ and the CeO_2_ particles in the metal can be swept over by inward growing α-Al_2_O_3_, (iii) the detection of cerium ions segregated to the GBs of the inner α-Al_2_O_3_, and (iv) precipitation of novel Ce_2_O_3_ particles along the twin boundaries in the outer γ-Al_2_O_3_. On these bases, we propose a model in the present work, which shows a scenario of a dynamic evolution of the ceria particles in the metal during oxidation. The model is helpful for better understanding of not only the REE on the alumina growth on the ceria-dispersed aluminide but also the concerned REE of the RE_x_O_y_ dispersions in other alumina formers. In addition, it is useful for getting insight into the alumina growth on the metals alloyed with REs, which can be preferentially oxidized into RE_x_O_y_ particles in the metals because the alloyed amounts of REs normally exceed their low solubility limits^1^,^2^,^3^,^7^,^23^,^25^,^26^,^51^.

## Results

### Ceria dispersion in aluminide before oxidation

The CeO_2_ particles used, which have a typical CaF_2_-type crystal structure (space group: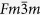) with the lattice spacing of *d*_(*200*)_ = 2.7 Å and *d*_(*111*)_ = 3.1 Å on a basis of HRTEM investigation and FFT diffraction (see supplementary material 1), are in a size range of 15–30 nm. The particles were co-deposited with Ni, forming a ~35 μm-thick Ni-based composite film, in which the CeO_2_ particles with the content of 3.5 wt.% are in general uniformly distributed, as viewed previously by using SEM^37^. After aluminizing, the Ni-CeO_2_ composite film was converted into a ~43 μm-thick alumina-forming δ-Ni_2_Al_3_^28^. The CeO_2_ particles are uniformly dispersed throughout the thickness of the aluminide on a basis of the electron probe microanalysis (EPMA)^37^.

### Ceria dispersion in aluminide and its evolution in alumina scale after oxidation

**Figure 1:**
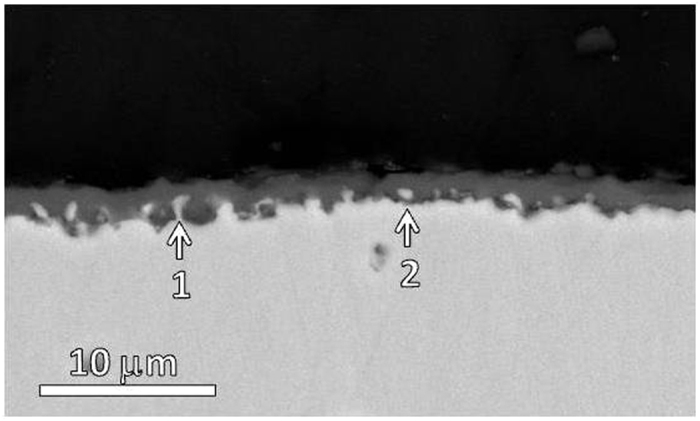
Cross-sectional SEM morphology of the nickel aluminide for 30 min oxidation at 1100 °C.

**Figure 2:**
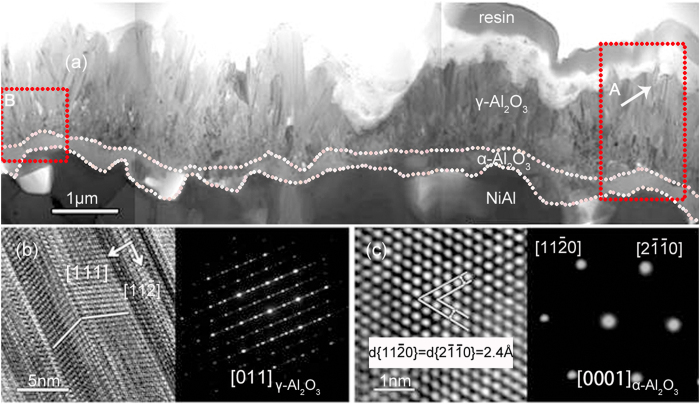
(**a**) Cross-sectional TEM overview of the alumina scale formed for 30 min oxidation at 1100 °C; (**b**,**c**) HRTEM images and corresponding SAED patterns of the outer and inner layers, respectively.

**Figure 3:**
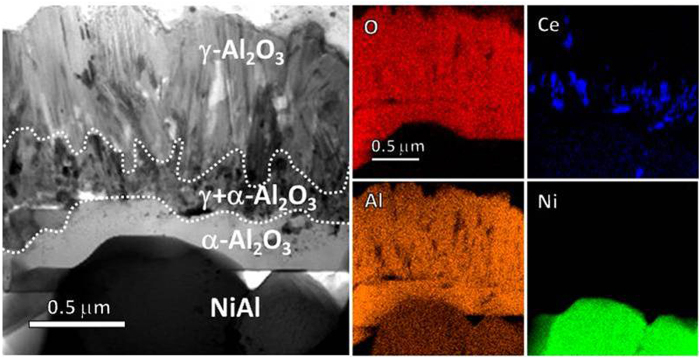
STEM BF image and EDS mappings of the “A”-framed area in Fig. 2(a).

**Figure 4:**
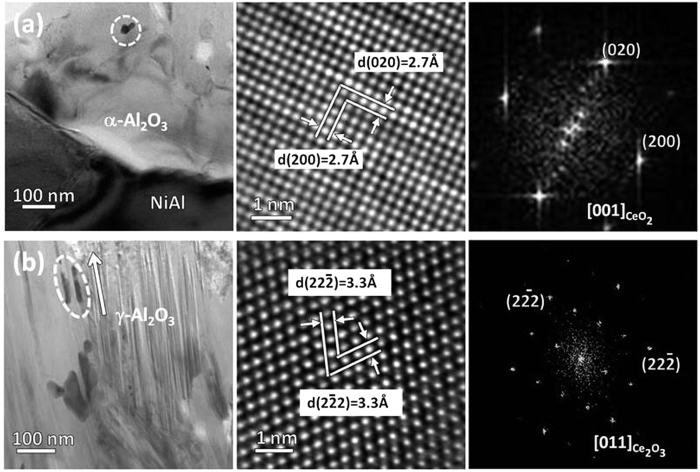
TEM BF and HRTEM images and corresponding FFT diffractions of the Ce-rich particle as circled in the (**a**) inner α-Al_2_O_3_ and (**b**) outer γ-Al_2_O_3_.

Figure 1 shows the cross-sectioned aluminide for 30 min oxidation at 1100 °C. The aluminide forms an alumina scale. The inward growth of the alumina, as suggested by the non-planar interface, leads the metal to be either partially (as indicated by 1) or fully (as indicated by 2) enclosed by the oxide. The alumina scale viewed under TEM as seen in Fig. 2(a) displays a double-layered structure. The outer needle-like platelets, which exhibit a high density of lamellar nanotwins with coherent boundaries when tilted to the [110] zone axis, are γ-Al_2_O_3_ as identified by HRTEM image and the corresponding SAED pattern in Fig. 2(b). Similar lamellar-twined structure has been observed in deformed fcc γ-grains of a single-phased austenitic steels^52^ and in Au nanocrystal-seeded Si and Ge nanowires^53^. This suggests that the growth of γ-Al_2_O_3_ platelets is controlled by outward diffusion of aluminum cations along the twin boundaries (TBs) in the [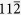] orientation. The oxide of the inner layer is α-Al_2_O_3_ as unveiled in Fig. 2(c). Between the γ-Al_2_O_3_ layer and the α-Al_2_O_3_ layer appears a γ- and α-mixed area as seen in Fig. 3. The γ- and α-Al_2_O_3_ grains are the smallest in the alumina scale and Ce-rich oxide nanoparticles (see the Ce X-ray mapping) can be sometimes observed. Similar Ce-rich oxide particles occur in the inner α-Al_2_O_3_ layer. They are CeO_2_ as identified in Fig. 4(a), displaying the shape and CaF_2_-type crystal structure similar to the original CeO_2_. The outer highly-twined γ-Al_2_O_3_ layer is also doped cerium-rich oxide particles, which as circled in the TEM BF image in Fig. 4(b) are seemingly elongated along the twinning orientation, with respect to the particle shape of the original CeO_2_. The 3.3 Å lattice spacing of both 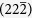 and 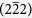 planes and (Mn_0.5_Fe_0.5_)_2_O_3_ (space group: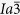)-similar structure (see HRTEM image and FFT diffraction in Fig. 4(b)) ascertain the particles as new Ce_2_O_3_ rather than the original CeO_2_.

**Figure 5:**
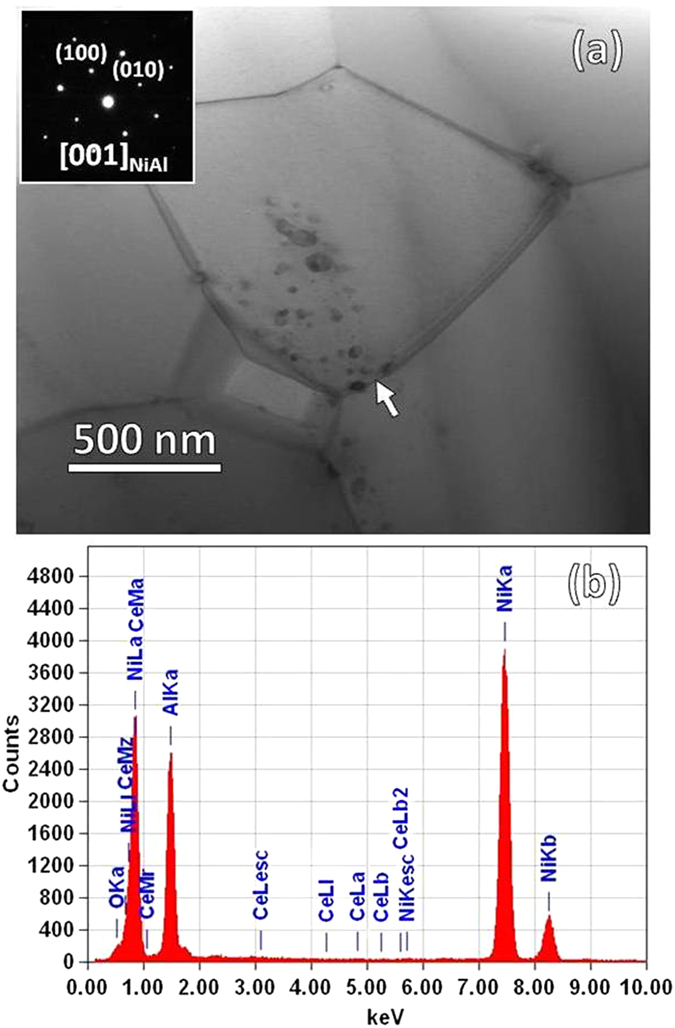
(**a**) STEM BF image of the aluminide for 30 min oxidation at 1100 °C; (**b**) an EDS spectrum of an arrowed spot between two close CeO_2_ particles at the aluminide grain boundary.

The aluminide has been degraded from δ-Ni_2_Al_3_ into β-NiAl due to the aluminum consumption by oxidation, as shown in the inserted SAED pattern in Fig. 5(a). The aluminide contains the nano-dispersions, which have been characterized to be original CeO_2_. No Ce was acquired around the CeO_2_ particles by the EDS detector with an incident beam spot size of 1.5 nm. Figure 5(b) shows an EDS result of a specific spot between two close CeO_2_ particles at the GBs, showing no acquisition of Ce atoms there.

**Figure 6:**
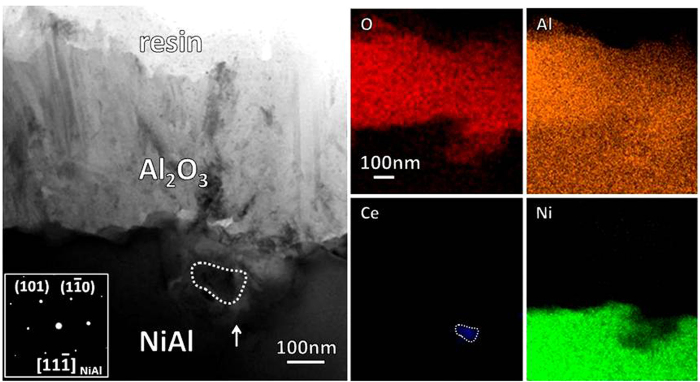
STEM BF image and corresponding elemental X-ray mappings of the cross-sectioned Al_2_O_3_ scale on the aluminide after 5 min oxidation at 1100 °C.

**Figure 7:**
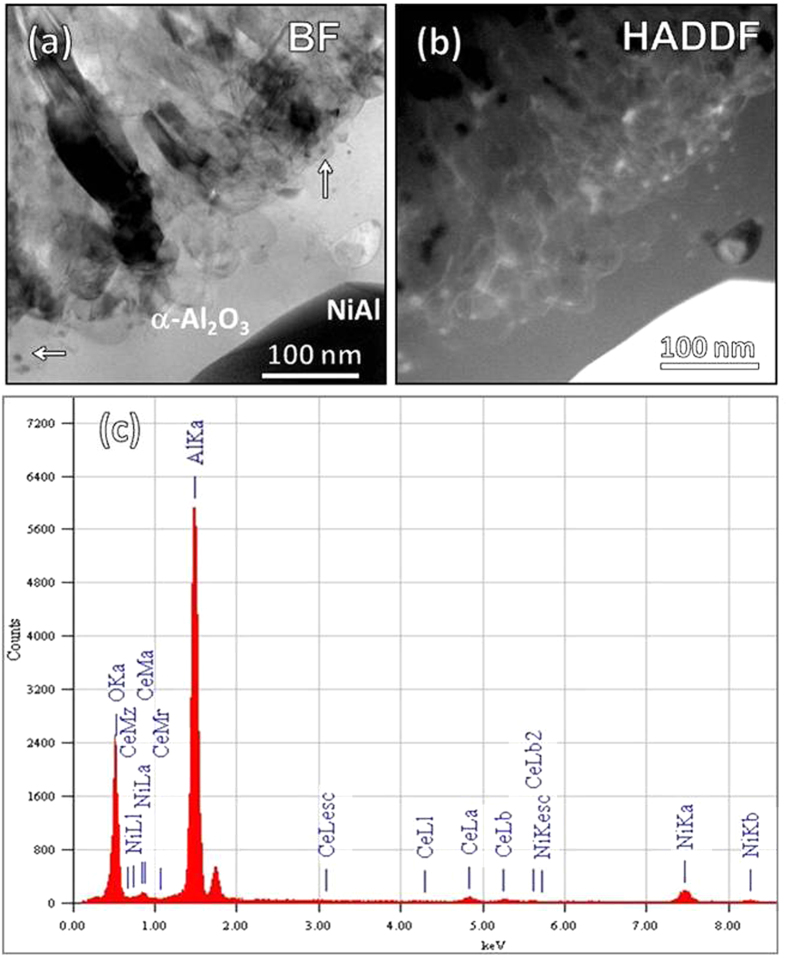
(**a**) Magnified STEM BF image of the “B”-framed area in Fig. 2(a); (**b**) corresponding HADDF image showing the segregation of the element with a lighter contrast at the alumina GBs; (**c**) EDS analysis identifying the segregated element as Ce.

The particles of the CeO_2_, as inert oxide in the metal, actually act as the immobile markers for the direction of the alumina growth. They occur in the fine-grained γ- and α-mixed area (Fig. 3), suggesting that the area corresponds to the surface zone of the original aluminide. The CeO_2_ particles in the α-Al_2_O_3_ layer arises from the inward growth of the oxide. To further clarify this, the alumina scale formed only for 5 min has been observed. A CeO_2_ particle which has been swept over by inward growing alumina is clearly seen in Fig. 6. In contrast, the Ce_2_O_3_ particles in the outer γ-Al_2_O_3_ layer should be newly precipitated. They can form, suggesting that there exist sufficient Ce cations which can be migrated from the inner α-Al_2_O_3_ layer. The larger-sized ions as the Ce cations here doped in the alumina TGOs are easily segregated to and then migrate outward along the GBs^15^,^17^. As shown in Fig. 7, the Ce segregation at the α-Al_2_O_3_ GBs can be clearly seen by using HADDF-STEM. The HADDF image presents the Ce segregated GBs presents as the lines with a light contrast similar to that of the CeO_2_ particles (as arrowed in the BF image), because Ce has a higher atomic number than Al. The EDS analysis indicates the GBs containing a mean content of ~0.4 at.% Ce. The Ce at the oxide GBs unlikely originates from its atoms in the aluminide, because the latter have not been acquired in the metal (Fig. 5). It convincingly arises from the segregation of cerium cations, produced by partial dissolution of the CeO_2_ particles incorporated in the α-Al_2_O_3_ layer. The dissolved Ce cations also experience the charge transformation from tetravalent to trivalent in the alumina scale, on a basis of the precipitation of Ce_2_O_3_ rather than original CeO_2_.

In sum, the TEM work presents several observations: (i) the aluminide during oxidation forms an alumina scale being composed of an inner α-Al_2_O_3_ layer and an outer γ-Al_2_O_3_ layer; (ii) the CeO_2_ dispersions are incorporated into the α-Al_2_O_3_ layer as the result of its inward growth; (iii) Ce ions segregates to the alumina GBs, and (iv) novel Ce_2_O_3_ particles are precipitated in the near surface of the γ-Al_2_O_3_ platelets.

## Discussion

**Figure 8:**
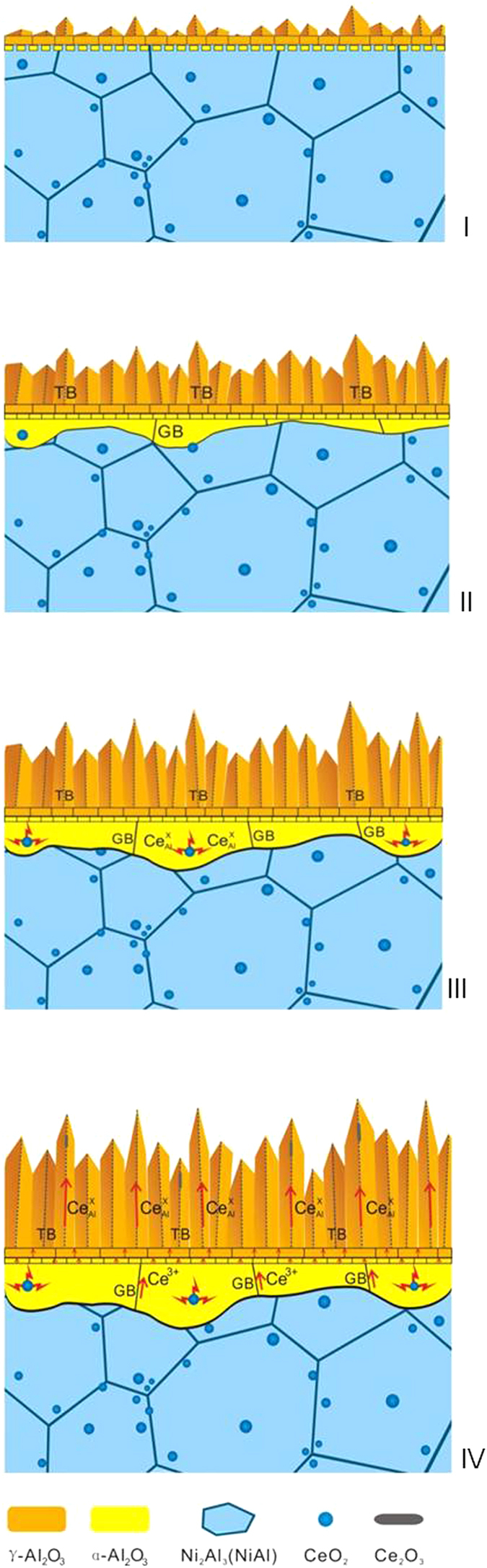
Schematic steps for the evolution of the CeO_2_ dispersion in the aluminide from being incorporated into inner Al_2_O_3_ scale to being precipitated as Ce_2_O_3_ in the outer Al_2_O_3_ scale.

The precipitation of new Ce_2_O_3_ in the outer γ-Al_2_O_3_ platelets demonstrates a series of evolution of the original CeO_2_ particles after they have been incorporated into the growing alumina, including their partial dissolution (since no evidence for such dissolution could be acquired in the aluminide (Fig. 5)), tetravalent-to-trivalent charge transformation and outward migration of the dissolved Ce cations. To unveil the dynamic evolution of the CeO_2_ dispersion in the aluminide, a model is schematically illustrated in Fig. 8 based on the TEM observations and interpreted below. The highly-twined γ-Al_2_O_3_ grains grow outward quickly on the aluminide at the onset of oxidation, and α-Al_2_O_3_ nucleates at the γ/aluminide interface soon after if not simultaneously (Step I). The γ-Al_2_O_3_ grains grow into needle-like platelets in the direction preferentially aligned with the [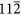] twinning orientation; in the meantime, the initially-formed α-Al_2_O_3_ grains gradually spread laterally and inward, sweeping over the CeO_2_ particles in the aluminide (Step II). α-Al_2_O_3_ exhibits an *n*-type behavior with the principal defect of oxygen vacancy 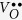 or free electron e′ (Vink-Kröger’s notation). The CeO_2_ partially dissolves into the α-Al_2_O_3_ lattice through the reaction


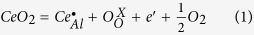


where 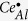 represents the quaternary-charged Ce cations and 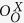 lattice oxygen. The 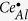 in the *n*-type oxide lattice can then trap an electron and transform to trivalent-charged cation 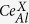 through the reaction


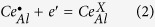


In combination of Eqs (1) and (2), the CeO_2_ particle dissolution in the alumina lattice can be expressed by


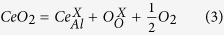


The reduction of CeO_2_ to Ce_2_O_3_ has been reported in the high temperature sintering of fine CeO_2_ particles^54^,^55^. The 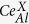 (1.02 Å with the coordination number of 6^56^) is larger than 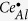 (0.87 Å with the same coordination number) in the ion size. Larger 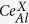 in the α-Al_2_O_3_ grains yields higher lattice misfit microstrain, which drives the trivalent cations to segregate to the α-Al_2_O_3_ GBs (Step III). Then, the segregated cations migrate from the α-Al_2_O_3_ layer to the γ-Al_2_O_3_ layer along the GBs in the α-Al_2_O_3_ and the lamellar TBs in the γ-Al_2_O_3_ platelets, under the driving force of the oxygen potential gradient across the oxide^15^,^17^. γ-Al_2_O_3_ is a *p*-type oxide with the principal defect of Al vacancy 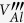 and electron hole 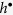. The TBs, although they are coherent, contain steps and kinks which can serve as the sinks for vacancy (as having been reported in^57^,^58^) like 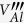 here. Steps and kinks in the lamellar TBs in the γ-Al_2_O_3_ can trap 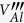. Once 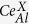 and 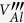 are both oversaturated there, Ce_2_O_3_ is precipitated (Step IV) through the reaction below,


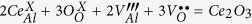


The 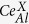 cations prefer to diffuse outward along the TBs, causing the Ce_2_O_3_ to be precipitated and elongated in the growth direction of the γ-Al_2_O_3_ platelets (Fig. 4(b)). The precipitates are easily observed in the γ-Al_2_O_3_ layer near the surface, because of higher 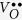 there which promotes the precipitation reaction.

The oxidation kinetics of alumina formers during 750–1200 °C is highly correlated with the diffusion of Al cations and O anions along the alumina GBs^30^. A decrease of 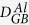 by the RE segregations to alumina GBs has been proposed to be the reason why the RE- and RE_x_O_y_−doped alumina formers have a lower oxidation rate^3^,^4^,^6^,^7^,^10^,^12^,^15^,^17^,^32^. As illustrated in Fig. 8, 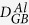 in the alumina layer here should be decreased when the Ce segregants outward migrate along the GBs in α-Al_2_O_3_ layer and the TBs in the γ-Al_2_O_3_ layer.

The model in Fig. 8 strongly suggests that the Ce segregants occur only when the CeO_2_ dispersoids in the aluminide have been swept over by the inward moving alumina/metals interface. In other word, the REE on the alumina growth for the CeO_2_ dispersion in the aluminide is intrinsically pertinent to the incorporation of the oxide particles into the alumina scale by its inward growth. This may be generalized to the REE of the other RE_x_O_y_ dispersions on alumina TGO growth. In addition, many alumina formers are alloyed with a RE instead of its oxide. Theoretically, it is possible that the RE in an alumina-forming metal at and below the equilibrium partial oxygen pressure of Al_2_O_3_/metal at the interface can be internally oxidized to form RE_x_O_y_. For example, Hf in an alumina-forming CoCrAlHf was internally oxidized into fine spherical HfO_2_ particles in an Al_2_O_3_/CoAl pack at 1200 °C^2^. The particle sizes of the formed RE_x_O_y_ highly depend on the synergistic effect of several factors, e.g., RE amounts and solubilities in metals, metal compositions and microstructures, alloying and oxidation temperatures^25^,^38^,^51^. The particles have not been highlighted previously, plausibly because they are sometimes as small as nano-sized particles. Because of a very low solubility limit in metals (e.g., only 0.01 wt % for Y in the FeCrAl alloys^51^), a RE overalloying is hard to avoid. Thus, RE-rich precipitates occur in metals^1^,^2^,^3^,^7^,^25^,^26^,^51^. They can be *in-situ* internally oxidized to form RE_x_O_y_ particles at the front of oxidation. The RE_x_O_y_ particles in the RE-alloyed metals, no matter whether they are formed by diffusional and non-diffusional oxidation of RE solute atoms and RE-rich precipitates, respectively, would not have the relative REE until they have been swept by the inward growing alumina.

In summary, the REE for the CeO_2_ particle dispersion in the nickel aluminide is firstly correlated with the inward growth of the inner α-Al_2_O_3_ in the TGO scale. The CeO_2_ dispersion, after being swept by the inward-growing α-Al_2_O_3_, partially dissolves producing tetravalent 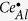. They then transfer to trivalent 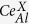 by capturing electrons and segregate to the GBs in the *n*-type α-Al_2_O_3_. The 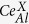 cations migrate outward along the GBs there and the TBs in the outer γ-Al_2_O_3,_ finally precipitating Ce_2_O_3_ near the surface. The segregation and migration of the Ce cations along the planar defects would obstruct the diffusion of the Al cations for the growth of the alumina TGO. The explanation may be generalized to the related REE of other RE_x_O_y_ dispersions in the alumina-forming metals. For the alumina formers alloyed with a RE, the RE, in the form of either solute atoms or RE-rich precipitates, is plausibly internally oxidized into RE_x_O_y_ particles below the TGO/metal interface. They may then affect the alumina growth in a manner to the CeO_2_ dispersion in the aluminide.

## Method

The CeO_2_ particles with a purity of 99.5%, a commercial product by Alfa Aesar company, were introduced to a nickel aluminide by using a two-step method^28^,^37^. First, the pure Ni samples with dimensions of 15×10×2 mm, after being abraded to a final 800 grit SiC paper, were electrodeposited with a Ni-CeO_2_ composite film from the CeO_2_-loaded nickel sulfate bath (150 g/l NiSO_4_·6H_2_O, 120 g/l C_6_H_5_Na_3_O_7_·2H_2_O, 12 g/l NaCl, 35 g/l H_3_BO_3_). A mechanical agitation was maintained to mitigate the particle agglomeration and sedimentation during electrodeposition, as illustrated by the setup^59^. Second, the samples were aluminized at 620 °C for 5 h using a halide-activated pack-cementation in a powder mixture of Al (particles size: ~75 μm) + 55 wt.% Al_2_O_3_ (~75 μm) + 5 wt.% NH_4_Cl in an Ar (purity: 99.99%) atmosphere. The characteristics of inward growth of the aluminide at the cementation temperature caused the CeO_2_ in the electrodeposited film to be trapped, forming a ceria-dispersed nickel aluminide coating on the sample surface. After being ultrasonically cleaned in acetone, the aluminized samples were ready for oxidation.

The samples were not placed into a muffle furnace for oxidation until it was heated up to 1100 °C. The ceria–dispersed nickel aluminide after oxidation were cross-sectioned for the scanning electron microscopy (SEM) investigations, and then ion sliced into thin foils by using techniques detailed elsewhere^32^, with the transparent areas desired for the TEM investigations under a JEOL 2100F TEM at 200 kV accelerating voltage, by using the techniques of bright field (BF) imaging and high resolution TEM (HRTEM) imaging and fast Fourier transformation (FFT) diffraction, scanning TEM (STEM) imaging as well as high-angle annular detector dark-field STEM (HADDF-STEM) imaging.

## Additional Information

**How to cite this article**: Wang, X. *et al*. The reactive element effect of ceria particle dispersion on alumina growth: A model based on microstructural observations. *Sci. Rep.* **6**, 29593; doi: 10.1038/srep29593 (2016).

## Supplementary Material

Supplementary Information

## References

[b1] Allam I. M., Whittle D. P. & Stringer J. The oxidation behavior of CoCrAl systems containing active element additions. Oxid Met. 12, 35–66 (1978).

[b2] Allam I. M., Whittle D. P. & Stringer J. Improvements in oxidation resistance by dispersed oxide addition: Al_2_O_3_-forming alloys. Oxid Met. 13, 381–401 (1979).

[b3] Ramanarayanan T. A., Raghavan M. & Petkovic-Luton R. The characteristics of alumina scales formed on Fe-based yttria-dispersed alloys, J. Electrochem Soc. 131, 923–931 (1984).

[b4] Luthra K. L. & Briant C. L. Mechanism of adhesion of alumina on MCrAlY alloys. Oxid Met. 26, 397–416 (1986).

[b5] Smeggil J. G. & Shuskus A. J. The oxidation behavior of some FeCrAlY, FeCrAl, and yttrium ion-implanted FeCrAl alloys compared and contrasted. J Vac Sci Technol. A4, 3577–3582 (1986).

[b6] Przybylski K. . Segregation of Y to grain boundaries in the Al_2_O_3_ scale formed on an ODA alloy J Electrochem Soc. 134, 3207–3208 (1987).

[b7] Ramanarayanan T. A., Ayer R., Petkovic-Luton R. & Lta D. P. The influence of yttrium on oxide scale growth and adherence. Oxid Met., 29, 445–472 (1988).

[b8] Jedliński J. & Borchardt G. On the oxidation mechanism of alumina formers Oxid. Met. 36, 317–337 (1991).

[b9] Pint B. A. & Hobbs L. W. Limitation on the use of ion-implantation for the study of the reactive element effect in β-NiAl. J Electrochem Soc., 141, 2443–2453 (1994).

[b10] Pint B. A., Garratt-Reed A. J. & Hobbs L. W. The reactive element effect in commercial ODS FeCrAl alloys. Mater High Temp. 13, 3–16 (1995).

[b11] Schumann E., Yang J. C., Graham M. J. & Rühle M. Segregation studies of oxidized Y and Zr doped NiAl. Mater. Corros. 46, 218–222 (1995).

[b12] Schumann E. The effect of Y-ion implantation on the oxidation of β-NiA1. Oxid Met. 43, 157–172 (1995).

[b13] Schumann E., Yang J. C., Rühle M. & Graham M. J. High-resolution SIMS and analytical TEM evaluation of alumina scales on beta-NiAl containing Zr or Y. Oxid Met. 46, 37–49 (1996).

[b14] Pint B. A., Tortorelli P. F. & Wright I. G. The oxidation behavior of ODS iron aluminides Mater Corros. 47, 663–674 (1996).

[b15] Pint B. A. Experimental observations in support of the dynamic segregation theory to explain the reactive element effect. Oxid Met. 45, 1–37 (1996).

[b16] Stott F. H. The oxidation of alumina-forming alloys. Mater Sci Forum 251–2, 19–32 (1997).

[b17] Pint B. A., Garratt-Reed A. J. & Hobbs L. W. Possible role of the oxygen potential gradient in enhancing diffusion of foreign ions on α-Al_2_O_3_ grain boundaries. J Am Ceram Soc. 81, 305–314 (1998).

[b18] Weinbruch S. . On the mechanism of high temperature oxidation of ODS superalloys: Significance of yttrium depletion within the oxide scales. Oxid Met. 51, 111–128 (1999).

[b19] Christensen R. J., Tolpygo V. K. & Clarke D. R. The influence of the reactive element yttrium on the stress in alumina scales formed by oxidation. Acta Mater. 45, 1761–1766 (1997).

[b20] Peng X., Li T. & Pan W. P. Oxidation of a La_2_O_3_-modified aluminide coating. Scripta Mater. 44, 1033–1038 (2001).

[b21] Nicholls J. R., Simms N. J. Chan W. Y. & Evans H. E. Smart overlay coatings – concept and practice. Surf Coat Technol. 149, 236–244 (2002).

[b22] Cueff R. . Oxidation behaviour of Kanthal A1 and Kanthal AF at 1173 K: Effect of yttrium alloying addition. Appl Surf Sci. 207, 246–254 (2003).

[b23] Pint B. A. Optimization of reactive-element additions to improve oxidation performance of alumina-forming alloys. J Am Ceram Soc. 86, 686–695 (2003).

[b24] Pint B. A. & Hobbs L. W. The Oxidation behavior of Y_2_O_3_-dispersed *β*-NiAl. Oxid Met. 61, 273–292 (2004).

[b25] Nijdam T. J. . On the microstructure of the initial oxide grown by controlled annealing and oxidation on a NiCoCrAlY bond coating. Oxid Met. 64, 355–377 (2005).

[b26] Nijdam T. J. & Sloof W. G. Effect of reactive element oxide inclusions on the growth kinetics of protective oxide scales. Acta Mater. 55, 5980–5987 (2007).

[b27] Naumenko D. . Correlation between the microstructure, growth mechanism, and growth kinetics of alumina scales on a FeCrAlY Alloy. Metall. Mater. Trans. A. 38A, 2974–2983 (2007).

[b28] Xu C., Peng X. & Wang F. Cyclic oxidation of an ultrafine-grained and CeO_2_-dispersed δ-Ni2Al3 coating. Corros Sci. 52, 740–747 (2010).

[b29] Choi H. J., Jedlinski J., Yao B. & Sohn Y. H. Transmission electron microscopy observations on the phase composition and microstructure of the oxidation scale grown on as-polished and yttrium-implanted β-NiAl. Surf Coat Technol. 205, 1206–1210 (2010).

[b30] Heuer A. H., Hovs D. B., Smialek J. L. & Gleeson B. Alumina scale formation: A new perspective. J Am Ceram Soc. 94, S146–S153 (2011).

[b31] Jedliński J. J. . The effect of alloyed and/or implanted yttrium on the mechanism of the scale development on β-NiAl at 1100 °C. Meter High Temp. 36, 59–69 (2012).

[b32] Peng X. . A fundamental aspect of the growth process of alumina scale on a metal with dispersion of CeO_2_ nanoparticles. Corros Sci. 53, 1954–1959 (2011).

[b33] Zhang H., Peng X. & Wang F. Fabrication of an oxidation-resistant β-NiAl coating on γ-TiAl. Surf Coat Technol. 206, 2454–2458 (2012).

[b34] Li D. Q. . Cyclic oxidation of β-NiAl with various reactive element dopants at 1200 °C. Corros Sci. 66, 125–135 (2013).

[b35] Jedliński J. . Development of oxide scale at 1,100 °C on Fe20Cr5Al alloy non-implanted and yttrium-implanted. Oxid Met. 79, 41–51 (2013).

[b36] Heuer A. H. . On the growth of Al_2_O_3_ scales. Acta Mater. 61, 6670–6683 (2013).

[b37] Tan X., Peng X. & Wang F. The mechanism for self-formation of a CeO_2_ diffusion barrier layer in an aluminde coating at high temperature, Surf Coat Technol. Surf. Coat. Technol. 224, 62–70 (2013).

[b38] Guo H. B. . Effect of Sm, Gd, Yb, Sc and Nd as reactive elements on oxidation behavior of β-NiAl at 1200 °C. Corros Sci. 78, 369–377 (2014).

[b39] Pint B. A., Unocic K. A. & Terrani K. A. Effect of steam on high temperature oxidation behaviour of alumina-forming alloys. Mater High Temp. 31, 28–35 (2015).

[b40] Pfeil L. B. Improvement in Heat-resisting Alloys, UK Patent, No. 459848 (1937).

[b41] Whittle D. P. & Stringer J. Improvements in high temperature oxidation resistance by additions of reactive elements or oxide dispersions. Philos. Trans. R. Soc. London Ser. **A** 295, 309–329 (1980).

[b42] Moon D. P. Role of reactive elements in alloy protection. Mater. Sci. Technol. 5, 754–764 (1989).

[b43] Jedlinski J. The influence of reactive elements on the high temperature oxidation behaviour of alumina – forming materials. Solid State Phenom. 21–22, 335 (1992).

[b44] Quadakkers W. J. & Singheiser L. Practical aspects of the reactive element effect. Mater. Sci. Forum. 369–372, 77–92 (2001).

[b45] Pint B. A. Progress in understanding the reactive element effect since the Whittle and Stringer literature review In Proc. John Stringer Symp. on High Temp. Corros. (eds Tortorelli P. F. & Hou P. Y. ) 27–37 (Ohio, 2003).

[b46] Hou P. Y. The reactive element effect – past, present and future. Mater. Sci. Forum. 696, 39–44 (2011).

[b47] Hirth J. P., Pieraggi B. & Rapp R. A. The role of interface dislocations and ledges as sources/sinks for point defects in scaling reactions. Acta Metall. Mater. 43, 1065–1073 (1995).

[b48] Pieraggi B. & Rapp R. A. Chromia scale growth in alloy oxidation and the reactive element effect. J. Electrochem. Soc. 140, 2844–2850 (1993).

[b49] Nakagawa T. . Yttrium doping effect on oxygen grain boundary diffusion in alpha-Al_2_O_3_. Acta Mater. 55, 6627–6633 (2007).

[b50] Gemming T., Nufer S., Kurtz W. & Rühle M. Structure and Chemistry of Symmetrical Tilt Grain Boundaries in α-Al_2_O_3_: I, Bicrystals with “Clean” Interface. J Am Ceram Soc. 86, 581–589 (2003).

[b51] Tien J. K. & Pettit F. S. Mechanism of oxide adherence on Fe-25Cr-4Al(Y or Sc) alloys. Metall Trans. 3, 1587–1599 (1972).

[b52] Yan F. K. . Deformation mechanism in an austenitic single-phase duplex microstructured steel with nanotiwned grains. Acta mater. 81, 487–500 (2014).

[b53] Davidson F. M. III. . Lamelar twinning in semiconductor nanowires. J Phys Chem C 111, 2929–2935 (2007).

[b54] Zhou Y. & Rahaman M. N. Effect of redox reaction on the sintering behavior of cerium oxide. Acta Mater. 45, 3635–3639 (1997).

[b55] Ozawa M. Effect of oxygen release on the sintering of fine CeO_2_ powder at low temperature. Script Mater. 50, 61–64 (2004).

[b56] Shoko E., Smith M. F. & McKenzie Ross H. Mixed valency in cerium oxide crystallographic phases: Determination of valence of the different cerium sites by the bond valence method. Physical Review B 79, 134108.1–134108.12 (2009).

[b57] Hsiao H. Y. . Unidirectional growth of microbumps on (111)-oriented and nanotwined copper. Science 336, 1007–1010 (2012). 22628648 10.1126/science.1216511

[b58] Liu T. C. . Eliminate Kirkendall voids in solder reactions on nanotwinned copper. Scripta Mater. 68, 241–244 (2013).

[b59] Peng X. Nanoscale assembly of high-temperature oxidation-resistant nanocomposites. Nanoscale 2, 262–268 (2010). 20644803 10.1039/b9nr00118b

