# Supplementary material for: The reactive element effect of ceria particle dispersion on alumina growth: A model based on microstructural observations
Source: Sci Rep. 2016 Jul 13;6:29593. doi: 10.1038/srep29593 (PMC4942799; doi:10.1038/srep29593)
Supplement: Supplementary Information [file srep29593-s1.pdf]

Supplementary information

The reactive element effect of ceria particle dispersion on alumina growth: A model based on microstructural observations

X. Wang[a,b], X. Peng, [a,b,*,z], X. Tan [b], F. Wang [b]

[a] School of Chemistry and Materials Science, University of Science and Technology of China, Hefei, 230026, China

[b] Laboratory for Corrosion and Protection, Institute of Metal Research, Chinese Academy of Sciences, Shenyang, 110016, China

* Corresponding author

Xiao Peng, Laboratory for Corrosion and Protection, Institute of Metal Research, Chinese Academy of Sciences, Shenyang, 110016, China

Tel: +86 24 23893753; fax: +86 24 23893624.

E-mail: xpeng@imr.ac.cn

SUPPLEMENTARY FIGURES:

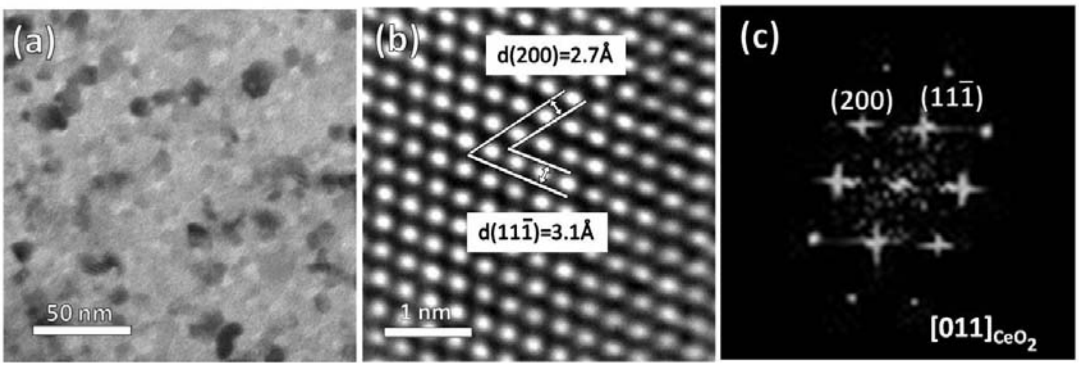

Supplementary Figure 1. Microstructure of as-purchased nanoparticles of CeO_2. (a) TEM BF and (b) HRTEM images, and (c) FFT diffraction pattern of as-purchased nanoparticles of CeO_2.
